# Supplementary material for: The interactome of intact mitochondria by cross-linking mass spectrometry provides evidence for coexisting respiratory supercomplexes
Source: Mol Cell Proteomics. 2017 Dec 8;17(2):216–32. doi: 10.1074/mcp.RA117.000470 (PMC5795388; doi:10.1074/mcp.RA117.000470)
Supplement: Supplemental Data [file supp_RA117.000470_133922_0_supp_23978_fzffwf.zip › spectra_annotation/mito_spectra_annotation/454-1-4-1-5-1.pdf]

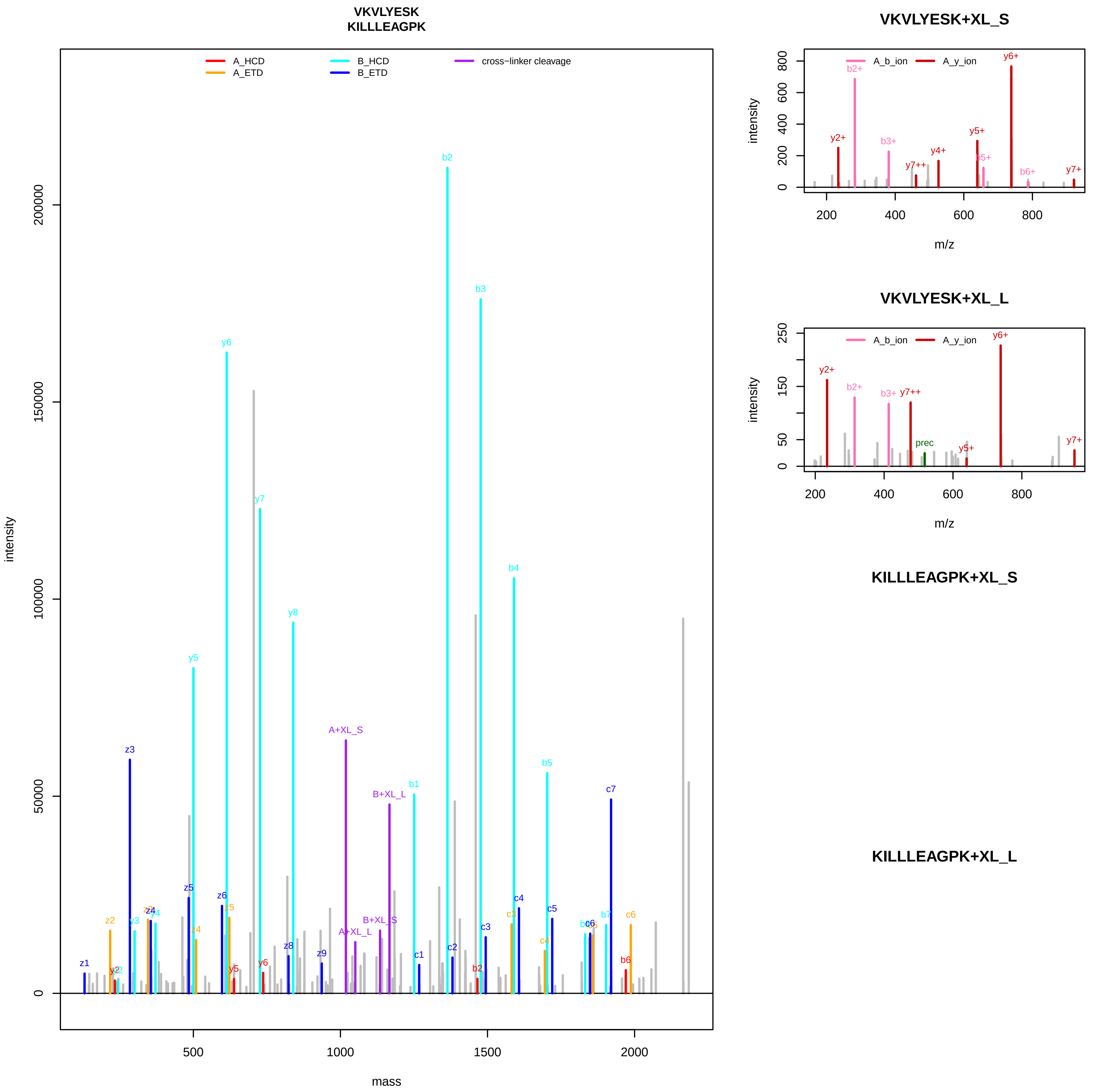
VKVLYESK
KILLLEAGPK
A_HCD
A_ETD
B_HCD
B_ETD
cross−linker cleavage
intensity
mass
0
50000
100000
150000
200000
500
1000
1500
2000
z1 z2 y2 y3 z3 z4 y4 z5 y5 z6 z5 z4 y5 y6 y6 y7 z8 y8 z9
A+XL_S A+XL_L B+XL_S B+XL_L
b1 c1 b2 c2 b2 b3 c3 b4 c3 c4 b5 c4 c5 b6 c6 b7 c7 b6 c6
VKVLYESK+XL_S
A_b_ion
A_y_ion
intensity
m/z
0
200
400
600
800
y2+ b2+ b3+ y7++ y4+ y5+ b5+ y6+ b6+ y7+
VKVLYESK+XL_L
A_b_ion
A_y_ion
intensity
m/z
0
50
150
250
200
400
600
800
y2+ b2+ b3+ y7++ prec y5+ y6+ y7+
KILLLEAGPK+XL_S
KILLLEAGPK+XL_L
